# Effect of zinc oxide nanoparticles (nZnO) on antioxidant defense, lignin metabolism and cadmium subcellular distribution in lettuce (*Lactuca sativa* L) under low-dose cadmium stress (hormesis)

**DOI:** 10.1371/journal.pone.0337953

**Published:** 2025-12-04

**Authors:** Feng Gao, Chengkui Qiang, Peilin Tao, Dan Cao, Wujian Feng

**Affiliations:** Department of Agriculture and Forestry Engineering, Xuzhou Vocational College of Bioengineering, Xuzhou, Jiangsu Province, China; Hainan University, CHINA

## Abstract

Most studies on cadmium (Cd) have focused on its toxicity at high concentrations, while its hormetic effects at low doses remain underexplored. Lettuce (*Lactuca sativa* L) is cultivated on a wide scale around the world, and its leaves continue to exhibit a high capacity for Cd accumulation at trace concentrations, thereby posing a significant threat to human health. This study investigated the role of foliar-applied zinc oxide nanoparticles (nZnO, 50 μmol l^-1^ and 100 μmol l^-1^) in mitigating low-dose cadmium (Cd) stress in lettuce. Exposure to 2.5 μmol L^-1^ Cd significantly enhanced lettuce growth, demonstrating a classic hormetic response. However, this growth stimulation was accompanied by Cd accumulation in leaves (4.45 mg kg^-1^ DW, 0.22 mg kg^-1^ FW), exceeding the FAO/WHO safety limit for edible vegetables (0.2 mg kg^-1^ FW). Foliar application of nZnO significantly reduced the levels of reactive oxygen species (ROS) and malondialdehyde (MDA) in lettuce. This was accompanied by increased activities of antioxidant enzymes and elevated endogenous hormone levels, which collectively contributed to enhanced lettuce growth. Furthermore, it was demonstrated that nZnO led to a substantial reduction in Cd accumulation in lettuce tissues. This reduction was attributed to an increase in total phenolic content and changes in the subcellular distribution and chemical forms of Cd. Our results indicate that foliar application of zinc oxide nanoparticles is a viable strategy for reducing Cd accumulation in lettuce and other leafy vegetables under Cd-induced hormesis, thereby ensuring food safety.

## Introduction

The intensification of agricultural and industrial activities has led to widespread cadmium (Cd) contamination of agricultural soils, particularly in developing countries. For instance, approximately 20% of the agricultural soils in China are contaminated with Cd [1]. Previous studies have shown that chronic Cd ingestion can cause deleterious effects on the kidneys and respiratory system, leading to chronic toxicity and severe damage to the immune system [2]. Although techniques like soil washing and phytoremediation are used to remove Cd from agricultural soils, they face limitations. These limitations are often attributable to the disruption of soil physicochemical properties and the protracted nature of the remediation process, which hinders their widespread adoption and implementation [3]. Therefore, exploring more effective methods to mitigate Cd accumulation in crops is crucial for ensuring food safety and promoting sustainable agriculture.

Cadmium exposure typically adversely affects plant morphology and physiology, consequently exerting a detrimental influence on their growth and productivity [4]. Notably, reproductive traits appear to be more susceptible to such influences in comparison to nutritional traits [5]. The relationship between Cd toxicity and its cumulative concentration in plants is well-documented, with substantial evidence showing that high doses of Cd have more pronounced effects on various aspects of plant growth. These effects include, but are not limited to, morphological, physiological and biochemical characteristics [6–9].Many past studies have focused on the effects of high Cd exposure on plants, e.g., high doses of Cd cause oxidative burst in plants [10], disrupt plant hormone homeostasis and uptake of essential elements [11,12], accelerates root lignification and retards root growth [13]. It is imperative to acknowledge that the elevated Cd exposure concentrations employed in numerous research endeavours are environmentally and ecologically implausible [14]. It is evident that when the concentration of Cd in the environment has not yet reached a level that would be considered to exceed the threshold of over-accumulation in plants, or prior to the accumulation of excessive Cd by the plants, the presence of low trace Cd has been shown to have an obvious stimulating effect on the growth and development of plants. This phenomenon, characterized by low-dose stimulation and high-dose inhibition, often described as an “inverted U-shaped” growth curve or hormesis, is considered a key factor in plant development [15,16]. For instance, low-dose Cd treatment has been shown to significantly stimulate the growth of lettuce [17], peanut [18], oilseed rape [19], and chard [20] seedlings.For some plant species, the hormesis effect can be leveraged as a strategy for the safe utilization of Cd-contaminated farmland [1]. For instance, low concentrations of Cd have been observed to enhance the yield of peppermint essential oil and promote improved growth performance of yellow essence, without affecting the safety of consuming yellow essence and using peppermint essential oil [1,21]. Nevertheless, in instances where certain plant species exhibit enhanced root and shoot performance in response to hormesis Cd toxicity, they concurrently accumulate Cd in their roots and shoots, rendering them potentially hazardous to both animal and human consumption [4,17,22].Previous studies have employed various technologies to mitigate Cd translocation in soil-plant systems, including crop rotation [23], screening of Cd-tolerant varieties [24], application of growth regulators, and utilisation of elemental nutrition [23]. While effective, these traditional approaches may face limitations; consequently, the incorporation of nanomaterials has emerged as a promising alternative strategy to enhance remediation efficiency. Among these technologies, nanoparticles (NPs) demonstrate considerable potential for safe crop production and remediation of heavy metal-contaminated soils, and their agricultural application is increasing [25].

It is well established that zinc is an essential element for a wide range of plant and human functions, and is required by all living organisms to maintain a wide range of physiological and metabolic functions [26]. However, many soils worldwide are facing zinc deficiency. In recent years, nZnO have been utilised as effective nano-fertilisers to enhance crop growth in zinc-deficient soils. The underlying rationale for this efficacy is attributable to the following properties: their minute size, high adsorption capacity, high surface-to-volume ratio, and high solubility in plants. Collectively, these characteristics facilitate enhanced zinc uptake by plants in comparison to conventional zinc fertilisers [27]. The efficacy of nZnO in enhancing the growth of various crops under Cd stress is well-documented [28]. This suggests that nZnO could serve as a promising candidate for mitigating Cd toxicity, thereby promoting safe agricultural practices. As demonstrated in earlier studies, the implementation of suitable concentrations of nZnO has been shown to impede the growth of wheat [29]. As indicated in the literature, concentrations of Cd in edible portions of rice [30], legumes [31], perilla [32], tomato [27], and chili pepper [33] have been documented [32].However, it has also been demonstrated that there is a synergistic effect between nZnO and Cd, which has the capacity to increase the concentration of Cd in green beans [34]. It has been determined that approximately 70% of the Cd ingested by humans in the surveyed peri-urban agricultural areas of central and southwest China originates from edible vegetables [35]. Green leafy vegetables have been observed to accumulate higher concentrations of Cd in the edible portion without exhibiting overt signs of toxicity in comparison to vegetables such as chili peppers and tomatoes [36]. As demonstrated in the study, leafy vegetables are typically consumed in their fresh state, thus constituting a primary source of Cd intake for humans [37]. This is concerning, as low-level Cd contamination is widespread in agricultural soils [38], and its toxicity can lead to Cd accumulation in leafy vegetables exceeding safe limits, thereby posing a serious risk to human health [39,40]. However, there is a paucity of research on using nZnO to mitigate Cd accumulation in green leafy vegetables specifically under the hormesis effect of Cd, a scenario that paradoxically promotes growth but compromises food safety. In order to resolve this issue, it is recommended that the impact of foliar spraying nZnO on the Cd content of green leafy vegetables be investigated.

Lettuce (*Lactuca sativa* L), a widely cultivated leafy green vegetable, exhibits a significant hormetic effect at low concentrations of cadmium, with the capacity to accumulate and stimulate growth [17]. The edible portion of lettuce has been found to possess a notable capacity for Cd enrichment, thus becoming an increasingly significant source of Cd intake for humans [41]. In view of the preceding discussion, the following hypothesis was formulated: that the administration of nZnO would result in a reduction of Cd accumulation in lettuce under conditions of toxic hormesis effects. Appropriate concentrations of nZnO have been demonstrated to possess the capacity to curtail Cd accumulation in plants through diverse application methodologies. In this study, a hydroponic experiment was employed to ensure the uniformity of the inter-root ion concentration and the controlled environment. The investigation encompassed a comprehensive array of physiological parameters, including hormone levels, antioxidant capacity, lignin metabolism, elemental content, and Cd accumulation. These parameters were analysed in lettuce under the hormesis effect of Cd toxicity, with the objective of elucidating the potential mechanisms of nZnO in mitigating Cd stress.

## Materials and methods

### Plant material and growing conditions

In this experiment, lettuce ‘Vanda’ was the less tolerant variety to Cd [14]. To ensure uniform germination, the seeds were soaked in distilled water for 8 hours. Subsequently, plump and uniform seeds were selected and placed in cultivation trays for the purpose of germination. After a period of 48 hours, the seeds that had germinated were transplanted into 72-hole cavity trays filled with vermiculite. These were then placed in an artificial climatic chamber with a relative humidity of 80%, a 24/18°C, a photoperiod of 16/8 hours, and a light intensity of 8,000 lx. The plants were irrigated with fresh water until they had developed two leaves. Subsequently, the seedlings were transferred to a plastic hydroponic box containing 1.6 L of Hoagland nutrient solution (25% ionic strength, pH=6.0), and the light intensity was gradually increased to 20,000 lx over a period of seven days. After this acclimatization period, the nutrient solution was modified to 50% ionic strength, and the light intensity was increased to 30,000 lx. The light intensity was increased to 30,000 lx. In order to ensure elemental availability in the nutrient solution and to avoid algal production, the nutrient solution was changed every three days. The composition of the Hoagland nutrient solution utilised in the study is delineated in Table 1.

**Table 1:** Composition of 1/2 Hoagland nutrient solution.

Components	Elemental concentrations
Liquid A	25 mmol·l^-1^ KNO_3_, 25 mmol·l^-1^ Ca(NO_3_)_2·_4H_2_O
Liquid B	7.5 mmol·l^-1^ MgSO_4_·7H_2_O, 5 mmol·l^-1^ NaH_2_PO_4_·2.5H2O
Trace elements	23 μmol·l^-1^ H_3_BO_3_, 4.75 μmol·l^-1^ MnSO_4_·H_2_O, 0.4 μmol·l^-1^ ZnSO_4_·5H_2_O, 0.15 μmol·l^-1^ CuSO_4_·5H_2_O, 0.008 μmol·l^-1^ (NH_4_)_6_Mo_7_O_2_·4H_2_O
Iron	0.04 μmol·l^-1^ EDTA·Na-Fe

### Experimental design and processing

The first experiment was undertaken using completely random design (CRD) with three replicates to assess the effects of different cadmium concentrations on lettuce growth. One week after transplanting the seedlings, add CdCl_2_ to each hydroponic box according to the treatment. The specific treatment is as follows: T0 (no treatment), T1 (exposure to 1 μmol l^-1^ CdCl_2_-2.5H_2_O), T2 (exposure to 2.5 μmol l^-1^ CdCl_2_-2.5H_2_O), T3 (exposure to 5 μmol l^-1^ CdCl_2_-2.5H_2_O) and T4 (exposure to 10 μmol l^-1^ CdCl_2_-2.5 H_2_O). The nutrient solution is replaced every four days to ensure that the concentration of Cd^2+^ in the solution remains stable. Plants were harvested after 28 days of growth for further analysis, and the optimal concentration for the cadmium hormesis effect was determined based on biomass and cadmium content.

The second experiment was also conducted using CRD, and three replicate groups were set up to evaluate the effects of low and high concentrations of nZnO. The treatments were as follows: CK (no treatment), Cd (exposure to 2.5 μmol l^-1^ CdCl_2_-2.5H_2_O), nZnO L (exposure to 2.5 μmol l^-1^ CdCl_2_-2.5H_2_O and foliar spraying of 50 μmol l^-1^ nZnO) and nZnO H (exposure to 2.5 μmol l^-1^ CdCl_2_–2.5 H_2_O and foliar sprayed with 100 μmol l^-1^ nZnO). nZnO (spherical, 30 ± 10 nm) and CdCl_2_-2.5H_2_O were purchased from Shanghai Aladdin Biochemical Science and Technology Co. Ltd (Shanghai, China). According to Huang et al. [42] the characterized nZnO nanoparticles had an average particle size of 36 nm (SEM) and 28 nm (TEM), consistent with the manufacturer’s specification of approximately 30 nm. Foliar application of nZnO solution commenced three days after Cd exposure. Before spraying, nZnO was dissolved in ultrapure water using ultrasound for 30 minutes. Spraying was performed until the leaves were moist but without water droplets. nZnO was foliar sprayed every 7 days, and three foliar sprays were performed in total. After 28 days of growth, fresh tissue samples from above and below ground parts of lettuce were collected and stored in an ultra-low temperature refrigerator at −80 °C for subsequent analysis.

Nutrient solutions containing Cd used in the experiment were collected in special containers and then handled by a professional company with the appropriate hazardous waste treatment qualifications.

### Determination of lettuce biomass

After harvesting, lettuce was washed with 5 mmol l^-1^ EDTA to remove residual metal elements on the surface, followed by thorough washing with deionized water and drying with absorbent paper, and the fresh weights of lettuce leaves and roots were measured using an electronic balance. Subsequently, the lettuce samples were dried in an oven at 60°C for 7 days for dry weight measurement.

### Determination of lettuce phytohormone content

A 0.5 g sample of lettuce tissue was rapidly ground into powder using liquid nitrogen, and then the endogenous hormone levels of auxin (IAA), Abscisic Acid (ABA), Gibberellin (GA_3_) and Zeaxanthin (ZT) in leaves and roots were measured using an elisa kit (Shanghai Cobre Bio-technology Co., Ltd., Shanghai, China). A SynergyTM LX multifunctional enzyme marker was used for the assay (Beijing Berten Instrument Co., Ltd., Beijing, China).

### Determination of malondialdehyde, H_2_O_2_, O_2_^-^content and antioxidant enzyme activities in lettuce

Malondialdehyde (MDA) content in lettuce tissues was determined using the 2-thiobarbituric acid (TBA) method [43], H_2_O_2_ content was measured using the 3,3-diaminobenzidine (DAB) method [44], and O_2_^-^ content was measured using the nitroblue tetrazolium (NBT) method [45].

Superoxide dismutase (SOD) activity in lettuce tissues was measured using the nitro blue tetrazolium (NBT) method described by Zhang et al [46]. For Peroxidase (POD) activity, it was measured using the guaiacol method described by Zhou et al [47]. Catalase activity (CAT) and ascorbate peroxidase (APX) activity were measured using the protocol of Khan et al [43].

### Determination of total phenols, lignin and lignin metabolizing enzyme activities in lettuce

Total phenol content in lettuce tissues was assessed using the acidified methanol method described by Xue et al [48], and total phenol content was expressed as gallic acid mg kg^-1^ FW. Lignin content was assessed using the method of Syros et al [49] and lignin content was expressed as OD 280 g^-1^ FW.

Phenylalanine deaminase (PAL), cinnamate-4-hydroxylase (C4H), 4-coumarate:coenzyme A ligase (4CL) and cinnamyl alcohol dehydrogenase (CAD) activities were determined according to the method of Chen et al [50].

### Analysis of elemental content and transporter factors in lettuce

Lettuce tissues were first dried in an oven at 80 °C for 72 h and ground to a fine powder using a pulverizer. Then it was digested with a solution of concentrated HNO_3_ and perchloric acid (4:1,v:v) on a graphite digestion furnace until the solution was colorless and transparent, after which it was diluted to 50 mL with ultrapure water. Determination of elemental contents in lettuce tissues The elemental contents of Cu, Fe, Mn, Zn, Ca, and Cd in digested samples were determined by inductively coupled plasma mass spectrometry (name of instrument, China) using the method described by He et al. [51]. Calculation of transport factor (TF) was performed according to the method described by An et al. [52] with the following equation:


$$TF=\frac{Cd\;in\;shoot}{Cd\;in\;root}$$

### Analysis of Cd and subcellular distribution of different chemical forms in lettuce tissues

The extraction and classification of various chemical forms of Cd were performed using the method described by Liu et al. [53]. Specifically, the sequential extraction procedure was as follows: 1) F_E_ (inorganic Cd) was extracted with 80% ethanol (v/v) at a ratio of 1:10 (tissue:extractant, g:mL) for 20 min at 25°C; 2) F_W_ (water-soluble Cd) was extracted with deionized water (1:10, g:mL) for 60 min at 25°C; 3) F_NaCl_ (pectin- and protein-bound Cd) was extracted with 1 M NaCl (1:10, g:mL) for 120 min at 25°C; 4) F_HAc_ (phosphate-chelated Cd) was extracted with 2% acetic acid (v/v, 1:10, g:mL) for 120 min at 25°C; 5) F_HCl_ (oxalate-bound Cd) was extracted with 0.6 M HCl (1:10, g:mL) for 120 min at 25°C; and 6) F_R_ (residual Cd) was digested with a HNO₃-HClO₄ mixture. All extracts were separated by centrifugation at 5,000 g for 10 min, and the Cd content in each fraction was determined by ICP-MS. The analysis of subcellular fractions of Cd was conducted in accordance with the extraction and classification methodology outlined by Jia et al. [54]. In summary, 0.2 g of frozen root tissue was thoroughly ground in pre-chilled 50 mM Tris-HCl extraction buffer (containing 250 mM sucrose and 1.0 mM dithiothreitol, pH = 7.5). The homogenate was centrifuged at 3,000 r·min^-1^ for 15 min, and the precipitate, consisting of cell walls and their fragments, was designated as the cell wall fraction (FII). The supernatant is further centrifuged at 15,000 r·min^-1^ for 30 min, with the resulting pellet and supernatant designated as the organ component (FII) and soluble component (FIII), respectively. All steps were performed at 4°C. The three fractions were dried and digested (specific methods detailed in 2.3.1) before being analyzed for cadmium content using ICP-MS.

### Statistical analysis

Experimental data were analyzed using IBM SPSS 20.0 (IBM, New York, USA). One-way analysis of variance (ANOVA) combined with Duncan’s multiple range test was used to assess significant differences between group means (p < 0.05).

The response between Cd content and phytohormones, MDA, reactive oxygen species, antioxidant enzymes, total phenols, lignin and lignin metabolizing enzymes in lettuce leaves under hormesis effect of foliar spraying of nZnO was comprehensively investigated by using normalized heat map matrix systematic analysis with hierarchical clustering, correlation coefficient analysis, and principal component analysis (PCA) and was analyzed and plotted by using Origin 2021 software.

## Results

### Effects of different cadmium concentrations on lettuce biomass and cadmium accumulation

**Fig 1:**
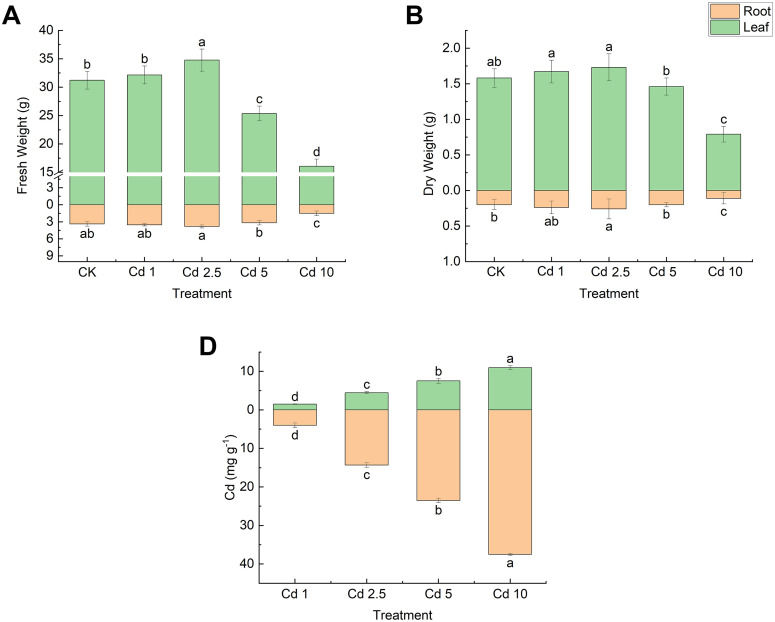
Effect of different concentrations of cadmium exposure on fresh weight (A), dry weight (B), and cadmium accumulation (C) of lettuce. Data are expressed as mean ± SE of three replicates. Bars labeled with different letters indicate significant differences according to Duncan’s test, the same blow.

The effects of Cd exposure on lettuce biomass were characterised by “low promotion and high suppression” (Fig 1). The leaf fresh weight and root dry weight increased significantly (p < 0.05) in the Cd 2.5 treatment in comparison with the CK treatment. In contrast, Cd 5 and Cd 10 treatments exhibited significant decreases (p < 0.05) in leaf fresh weight, root fresh weight, leaf dry weight and root dry weight in comparison with the CK treatment, while the Cd 1 treatment did not demonstrate any significant changes in relation to the CK treatment. As shown in Fig 1, a positive correlation was found between Cd accumulation in lettuce leaves and roots and the exposure concentration. However, it was noted that the Cd content in leaves of the Cd 2.5 treatment (4.45 mg kg-1 DW, 0.22 mg kg^-1^ FW) had surpassed the limit value stipulated by China (0.2 mg kg^-1^ FW). Consequently, it can be deduced that the incorporation of 2.5 μmol l^-1^ of Cd manifested a substantial toxic hormesis effect on lettuce.

### Effect of zinc oxide nanoparticles on lettuce fresh weight and endogenous hormones

**Fig 2:**
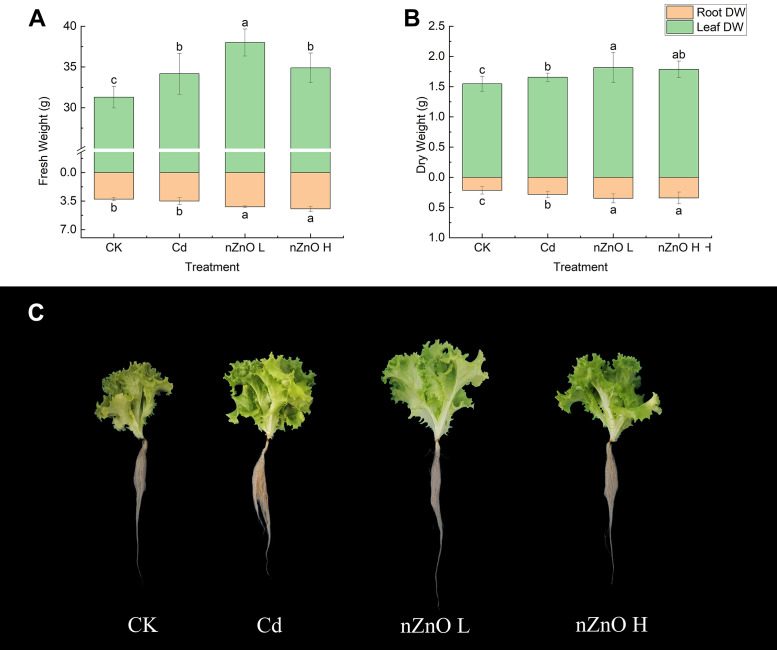
Effect of zinc oxide nanoparticles on fresh weight (A), dry weight (B) and morphology (C) of lettuce tissues.

Lettuce plants exposed to 2.5 μmol·l^-1^ Cd for 28 days were analysed by foliar spraying with low and high concentrations of nZnO treatments (50 μmol·l^-1^ nZnO L and 100 μmol·l^-1^ nZnO H), and the results are shown in Fig 2. Consistent with the results from section 3.1, the Cd treatment significantly increased the fresh and dry weights of leaves and roots compared to the CK treatment. The present study demonstrated that increased nZnO L treatment resulted in a positive effect on the growth of lettuce, and significantly increased (p < 0.05) the fresh and dry weights of leaves and roots in comparison with the Cd treatment. In contrast, while the nZnO H treatment did not significantly affect leaf biomass compared to the Cd treatment, it significantly increased the fresh and dry weights of the roots.

**Fig 3:**
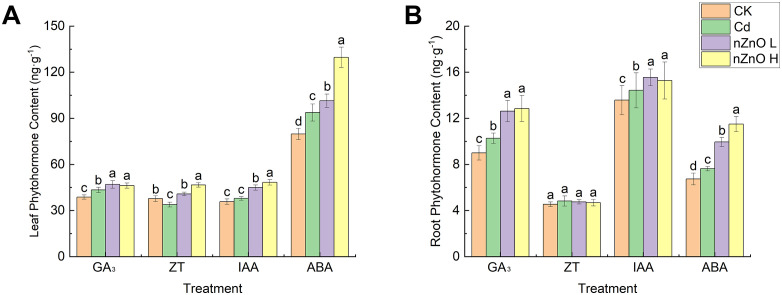
Effect of zinc oxide nanoparticles on endogenous hormone (GA_3_, ZT, IAA and ABA) content in leaves (A) and roots (B) of lettuce.

The levels of GA_3_, ZT, IAA and ABA in lettuce tissues were determined in order to assess the effect of nZnO on endogenous hormone homeostasis in lettuce under Cd-induced hormesis. Overall, the concentrations of endogenous hormones in Cd-treated plants were significantly higher (p < 0.05) than those in CK-treated plants, with the exception of the significantly lower (p < 0.05) ZT content in leaves (Fig 3A). The concentrations of GA_3_ and ABA in leaves and the concentrations of GA_3_, IAA and ABA in roots were also lower in the Cd-treated plants. In comparison with the Cd treatment, the nZnO L and nZnO H treatments resulted in a significant increase (p < 0.05) in the concentrations of GA_3_, ZT, IAA, and ABA in leaves and GA_3_, IAA, and ABA in roots. However, the concentration of ZT in roots did not change significantly.

### Effect of zinc oxide nanoparticles on reactive oxygen species, membrane lipid peroxidation and antioxidant enzyme activities in lettuce under Cd-induced hormesis

**Fig 4:**
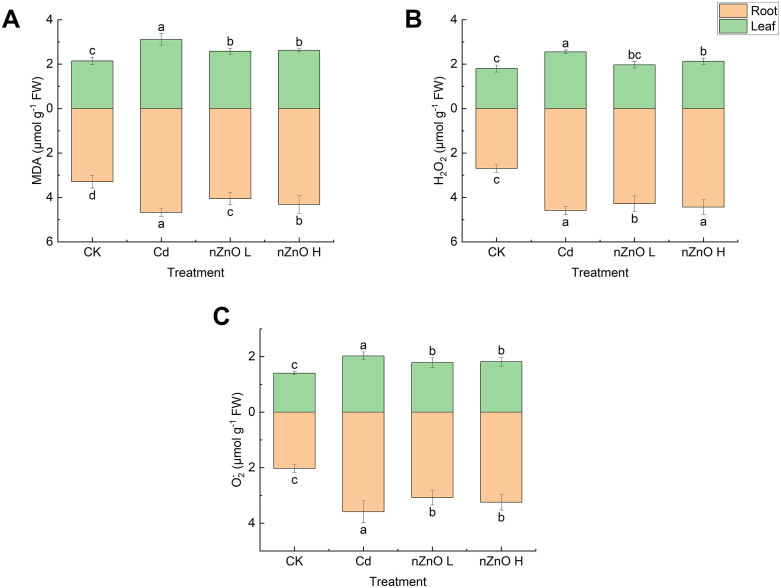
Effect of zinc oxide nanoparticles on MDA (A), H_2_O_2_ (B) and O_2_^-^ (C) contents of lettuce leaves and roots.

To assess how nZnO L alleviated oxidative stress under this hormetic condition, we analysed the concentrations of MDA, H₂O₂, and O₂^-^ in leaves and roots (Fig 4). Cd treatment significantly increased (p < 0.05) the concentrations of MDA, H₂O₂, and O₂⁻ in both leaves and roots relative to the CK. However, the nZnO L treatment markedly reduced the levels of these oxidative stress markers compared to the Cd-only treatment. Furthermore, the nZnO H treatment demonstrated significant reductions in MDA, H₂O₂ and O₂ concentrations in leaves, as well as MDA and O₂ concentrations in roots. However, no significant changes in H₂O₂ concentrations in roots were observed (Fig 4B).

**Fig 5:**
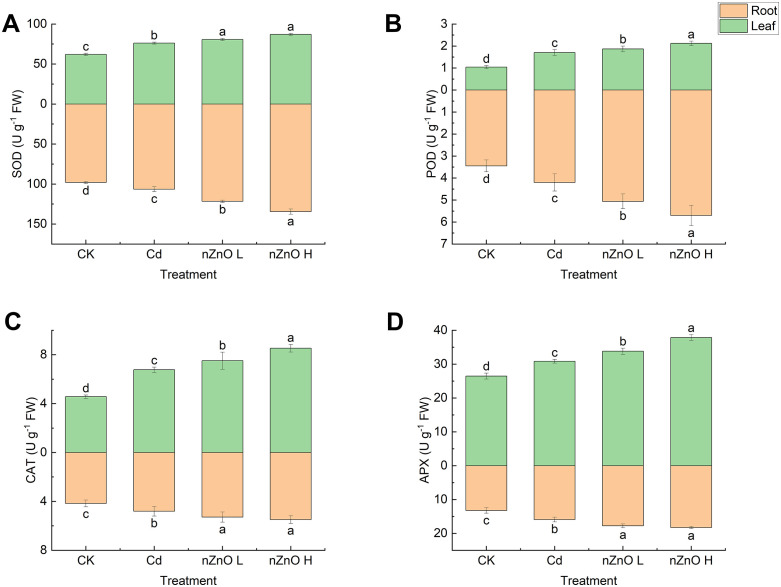
Effect of zinc oxide nanoparticles on SOD (A), POD (B), CAT (C) and APX (D) activities of lettuce leaves and roots.

It is noteworthy that exposure to Cd alone had a significant effect on the antioxidant enzyme activities of lettuce (Fig 5).Treatment with Cd resulted in a substantial increase (p < 0.05) in the enzyme activities of SOD, POD, CAT and APX in both leaves and roots, in comparison with the CK treatment. In comparison with Cd treatment, nZnO L and nZnO H treatments resulted in a significant increase (p < 0.05) in the enzyme activities of SOD, CAT and APX in both leaves and roots. Notably, the nZnO H treatment exhibited a more pronounced increase in SOD and POD activities (Fig 5A, 5B).

### Effect of zinc oxide nanoparticles on total phenols, lignin and lignin metabolizing enzyme activities in lettuce

**Fig 6:**
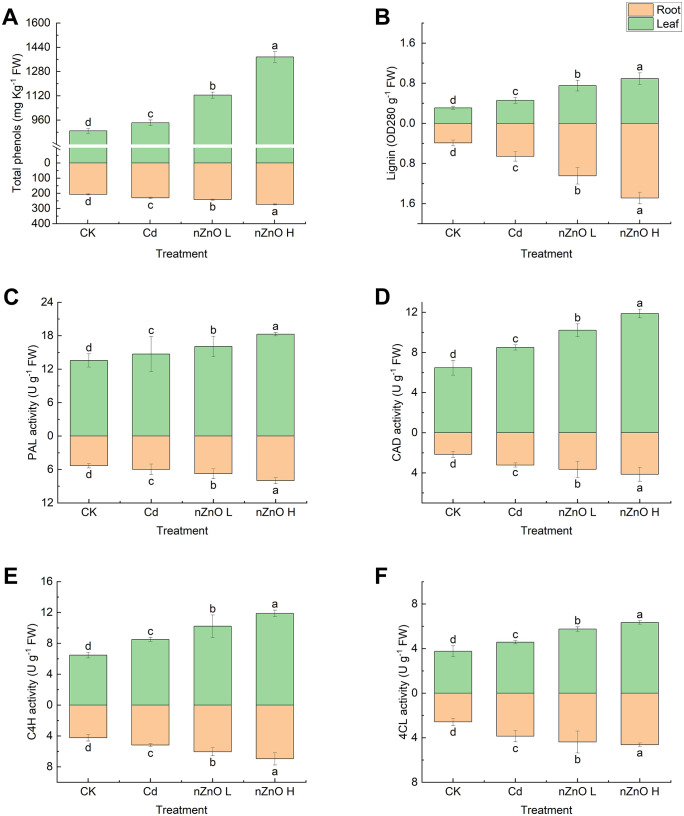
Effect of zinc oxide nanoparticles on Total phenols content (A), lignin content (B), PAL (C), CAD (D), C4H (E) and APX (F) activities of lettuce leaves and roots under the hormesis effect of Cd.

The effects of the treatments on the total phenol content, lignin content, PAL, CAD, C4H and APX activities of lettuce tissues were compared (Fig 6). Cd exposure significantly induced (p < 0.05) the phenylpropanoid pathway, manifesting as elevated levels of total phenols, lignin, and the activities of key biosynthetic enzymes (PAL, C4H, 4CL, CAD) in both leaves and roots compared to the CK treatment. Foliar application of nZnO, particularly at the higher concentration (nZnO H), further amplified this response, resulting in a dose-dependent augmentation of these metabolites and enzymes beyond the levels observed in the Cd-only treatment. This suggests nZnO not only mitigates Cd stress but actively enhances the synthesis of these protective compounds.

### Effect of zinc oxide nanoparticles on the elemental content of lettuce tissues

**Fig 7:**
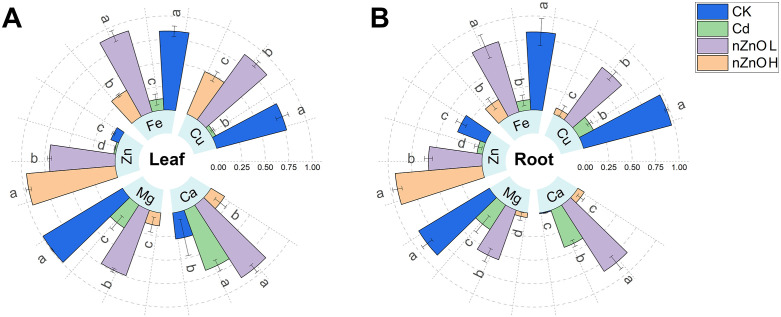
Effect of zinc oxide nanoparticles on elemental content of lettuce leaves (A) and roots (B) under the hormesis effect of cadmium toxicity. The data were maximum-minimum normalized so that the resultant values were mapped between [0 - 1].

Cadmium and nZnO treatments significantly altered mineral nutrient homeostasis (Fig 7, Table 2). Cd stress alone disrupted nutrient homeostasis, reducing accumulation of Cu, Fe, Mg, and Zn while increasing Ca content compared to the CK. Foliar application of nZnO exhibited a concentration-dependent effect: the lower concentration (50 μmol·l^-1^, nZnO L) mitigated Cd-induced deficiencies by enhancing the contents of Cu, Fe, Mg, and Zn, whereas the higher concentration (100 μmol·l^-1^, nZnO H) was less effective in restoring most nutrients and further significantly reduced Ca content, indicating a potential shift from nutritional supplementation to ionic antagonism at elevated concentrations.

**Table 2:** Effect of zinc oxide nanoparticles on the elemental content of lettuce tissues under the hormesis effect of Cd. Values are presented as mean ± SE (n = 3). Different lowercase letters within a column indicate significant differences according to Duncan’s test (p < 0.05). The percentage change for nZnO treatments, shown in brackets, is calculated relative to the Cd-only treatment.

Leaf	Cu (mg Kg^-1^)	Fe (mg Kg^-1^)	Mg (mg Kg^-1^)	Zn (mg Kg^-1^)	Ca (mg Kg^-1^)
CK	33.67 ± 1.09a (-)	115.29 ± 2.54a (-)	6564.89 ± 57.02a (-)	104.66 ± 3.62c (-)	16374.16 ± 108.19b (-)
Cd	27.17 ± 0.26c (-)	81.3 ± 2.79c (-)	5855.02 ± 89.45c (-)	88.13 ± 1.76d (-)	17678.29 ± 640.80a (-)
nZnO L	25.72 ± 0.35a (−5.3%)	118.22 ± 2.89a (+45.4%)	6310.44 ± 23.08b (+7.8%)	248.49 ± 5.13b (+182.0%)	17108.42 ± 206.87a (−3.2%)
nZnO H	22.12 ± 0.39b (−18.6%)	90.66 ± 0.74b (+11.5%)	5695.03 ± 82.46c (−2.7%)	306.77 ± 6.67a (+248.2)	15985.12 ± 156.92b (−9.6%)
Root					
CK	7.09 ± 0.50a (-)	747.15 ± 18.95a (-)	4698.93 ± 33.10a (-)	342.56 ± 6.34d (-)	9053.43 ± 136.35c (-)
Cd	5.83 ± 0.78c (-)	652.35 ± 7.69b (-)	4404.53 ± 58.99bc (-)	313.53 ± 4.77c (-)	10001.84 ± 83.18b (-)
nZnO L	5.65 ± 0.15b (−12.7%)	740.96 ± 17.29a (−10.2%)	4535.31 ± 39.50b (+5.7%)	371.65 ± 5.07b (+155.7%)	10968.39 ± 191.07a (+7.9%)
nZnO H	4.78 ± 0.57c (−22.1%)	665.5 ± 9.58b (−21.0%)	4273.16 ± 22.51c (−2.9%)	411.62 ± 5.78a (+207.5)	9211.62 ± 88.85c (−4.2%)

### Effect of zinc oxide nanoparticles on cadmium content and transport in lettuce tissues

**Fig 8:**
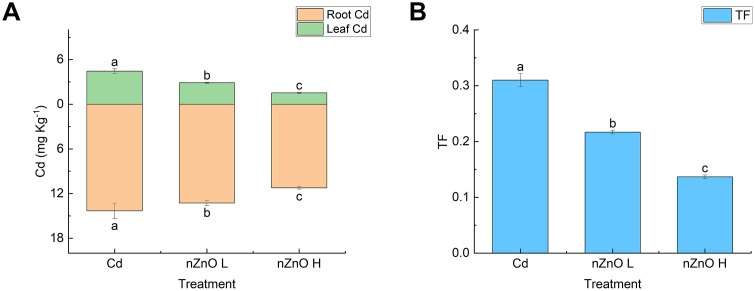
Effect of zinc oxide nanoparticles on cadmium content (A) and transport coefficient (B) in leaves and roots of lettuce.

Foliar spraying of nZnO was linked to a significant reduction (p < 0.05) in both Cd content and its translocation factor (TF) from roots to shoots (Fig 8). This suggests that nZnO may not only impede Cd uptake but also its translocation within the plant, thereby effectively protecting the edible aerial parts. The treatments of lettuce plants with nZnO L and nZnO H resulted in a substantial decrease in the content and transport coefficients of Cd in the leaves and roots when compared to the Cd treatments. Notably, the effect of the high concentration of nZnO H was more pronounced.

**Fig 9:**
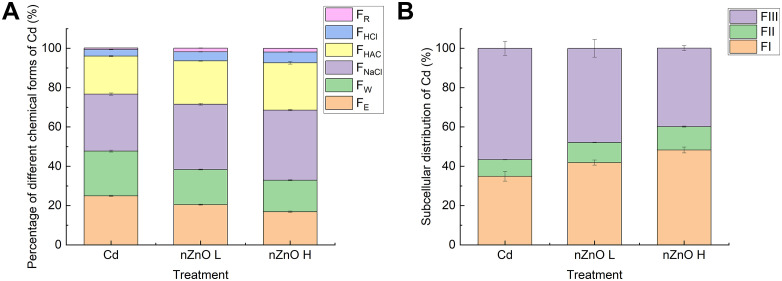
Subcellular distribution (FI: Cell wall; FII: Organelles; FIII: Soluble fraction) and chemical forms (F_NaCl_: pectin- and protein-bound Cd; F_HAc_: phosphate-chelated Cd; F_HCl_: oxalate-bound Cd; F_R_: residual Cd) of Cd in lettuce roots and leaves under different treatments.

The mitigation of Cd toxicity by nZnO coincided with notable changes in Cd chemical forms and subcellular distribution. As illustrated in Fig 9A, nZnO treatments significantly decreased the proportion of highly toxic and mobile forms (F_E_ and F_W_) while increasing the more stable and insoluble forms (F_NaCl_, F_HAC_, F_HCl_ and F_R_). It was demonstrated that a high concentration of nZnO H treatment was more effective than a low concentration of nZnO L treatment. Furthermore, at the subcellular level (Fig 9B), nZnO application increased the capacity of the root cell wall (FI) to sequester Cd, thereby reducing its proportion in the more sensitive organelle fraction (FII). This shift towards inactive forms and compartments is a key mechanism by which nZnO reduces Cd phytotoxicity.

### Comparison of biomass, hormone, oxidative stress and lignin metabolism correlations of lettuce tissues under the hormesis effect of cadmium toxicity by zinc oxide nanoparticles

**Fig 10:**
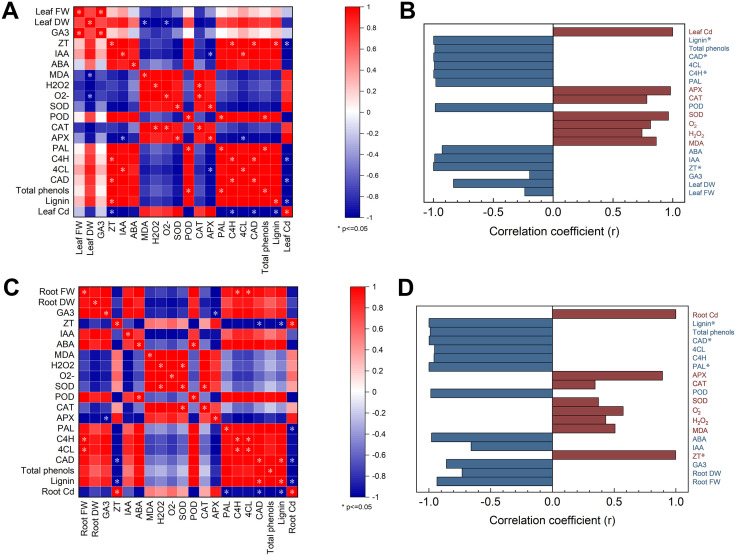
Effects of different concentrations of Cd on the microstructure of lettuce. (A,B) aboveground parts of lettuce, (C,D) roots.

In the context of lettuce plants exposed to Cd, the response to Cd divided into two groups, with positive and negative correlations, as revealed by the analysis of the normalized heat map matrix of roots and leaves. A comparative analysis of factors associated with Cd content was conducted, which revealed positive correlations between antioxidant enzyme activities (APX, SOD, CAT), reactive oxygen species (H₂O₂, O₂^-^) and MDA in leaves, lignin metabolism (lignin, total phenols, CAD, 4CL, C4H, PAL), endogenous hormones (ABA, IAA, ZT, GA₃) and biomass. Conversely, a negative correlation was observed (Fig 10A, 10B). A comparative analysis of factors associated with Cd content in roots was found to be similar to that in leaves; however, ZT content demonstrated a significant positive correlation (Fig 10C, 10D).

### Normalized thermogram response of lettuce tissue to the hormesis effect of cadmium toxicity by zinc oxide nanoparticles

**Fig 11:**
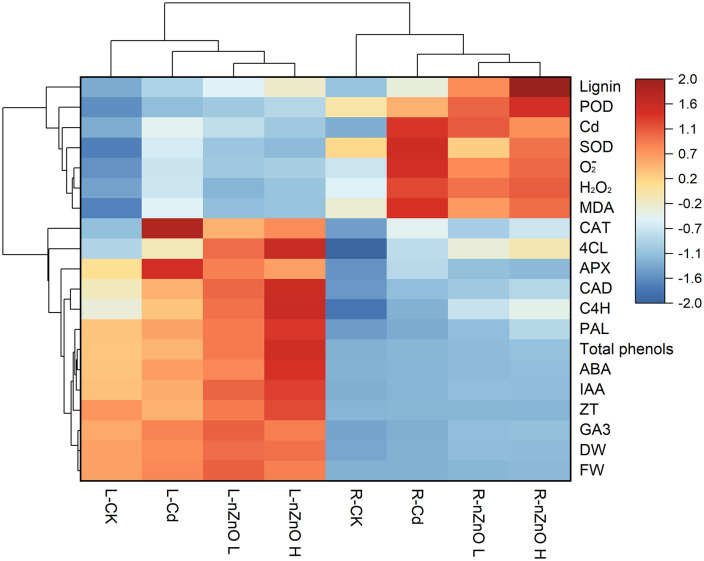
Normalized heat map matrices of biomass, hormones, oxidative stress and lignin metabolism observed in lettuce tissues under hormesis effects of Cd in roots and leaves treated with different nZnO levels.

The response of lettuce tissues to nZnO treatment under the hormesis effect of Cd was assessed using a normalized heat map matrix with hierarchical clustering (Fig 11). It was found that nZnO treatment and Cd treatment resulted in different responses between leaves and roots. In both leaves and roots, all the indices were elevated in the Cd treatment in comparison to the CK treatment. The nZnO treatment resulted in a reduction of oxidative stress (SOD, APX, CAT, MDA, H_2_O_2_, O_2_^-^) and an enhancement of indices related to endogenous hormones (IAA, ABA, ZT, GA_3_) and lignin metabolism (total phenols, POD, PAL, 4CL, CAD, 4CH, lignin). In general, the normalized responses of CAT, 4CL, APX, CAD, C4H, PAL, total phenols, ABA and GA_3_ were found to be higher in leaves than in roots. Conversely, the normalized responses of lignin, POD, SOD, O_2_^-^, H_2_O_2_ and MDA were found to be higher in roots than in leaves.

### Principal component analysis of lettuce tissues under the hormesis effect of cadmium toxicity by zinc oxide nanoparticles

**Fig 12:**
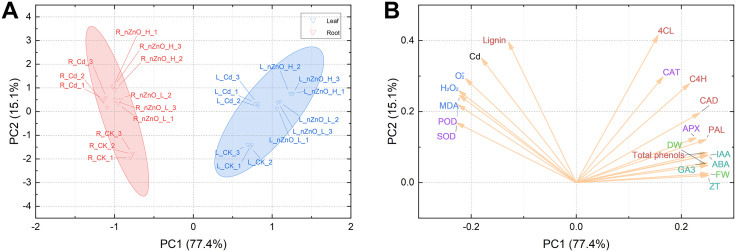
Score plots of principal component analysis (PCA) for biomass, hormones, oxidative stress and lignin metabolism of lettuce roots and leaves under the hormesis effect of Cd at different nZnO levels (A), and (B) loadings of biomass, hormones, oxidative stress and lignin metabolism of lettuce roots and leaves at different nZnO levels.

The combined response of lettuce tissues to nZnO treatments under Cd-induced hormesis was assessed by using score plots employing principal component analysis (Fig 12A), with PC1 and PC2 values accounting for 77.4% and 15.1% of the total x- and y-variance, respectively. Significant segregated clustering was detected between lettuce roots and leaves: in leaves and roots, Cd treatment, nZnO L treatment and nZnO H treatment were significantly separated compared to CK treatment. In addition, as the applied nZnO concentration increased, the responses of leaves and roots were increasingly far away from those of the Cd treatment, and the effects were more pronounced in leaves than in roots. These results suggest that the modification of the excitotoxic effect of Cd on lettuce is dependent on the concentration of nZnO. Associations between response variables were assessed using principal component analysis load plots (Fig 10B). There were two main associations: (a) Cd content, lignin, SOD, POD, H_2_O_2_, O_2_^-^ and MAD were closely related; (b) 4CL, CAT, C4H, CAD, APX, PAL, Total phenols, GA_3_, IAA, ABA, ZT, FW and DW were closely related.

## Discussion

Cadmium (Cd), a non-essential element for plants, is widely recognized as one of the most toxic heavy metals. A considerable number of studies have demonstrated that high doses of Cd stress can inhibit plant growth. However, a growing body of research has revealed that low-dose Cd treatments can stimulate growth, exhibiting toxic hormesis effects. However, this growth stimulation often coincides with significant accumulation of Cd in edible tissues, creating a critical food safety challenge that remains understudied. For instance, low-dose Cd treatment significantly stimulated the growth of peanut seedlings [18], and oilseed rape has been shown to respond with increased plant height, primary root length, and dry weight [19]. As demonstrated by Lin et al. [55] and Jia et al. [56], low doses of Cd have been shown to significantly increase plant height and biomass of Honeysuckle. In our study using lettuce, a similar pattern of “low-dose stimulation and high-dose inhibition” was observed. The Cd treatments at 1 μmol l⁻¹ and 2.5 μmol l⁻¹ stimulated growth and increased the fresh and dry weights of leaves and roots, while the Cd treatments at 5 μmol l^-1^ and 10 μmol l^-1^ significantly suppressed the growth of lettuce, with an initial increase and subsequent decrease. The growth of lettuce was significantly inhibited by the Cd treatments of 2.5 and 10 μmol l^-1^, showing an increasing and then decreasing trend (“inverted U-shape”). The increase in dry and fresh weight of lettuce in the Cd 2.5 treatment was the most significant, and the Cd content in leaves exceeded the limit value stipulated in China.

Endogenous hormones are known to play a key role in regulating plant responses to Cd exposure. Previous studies on the relationship between Cd and plant hormones have indicated that GA_3_ [57], ABA [58], ZT [59], and IAA [60] have the capacity to reduce the accumulation of Cd and oxidative stress in a variety of plants. Furthermore, changes in the levels of endogenous hormones in plant tissues have been observed to tend to promote plant growth. The concurrent significant promotion of plant growth (Fig 2) and modulation of endogenous phytohormones (Fig 3) under low-dose Cd stress suggest a potential role for hormonal signaling in mediating the hormetic response, as reported in other species [61,62]. Foliar application of nZnO further amplified this hormonal shift. While our physiological data support this association, the precise molecular mechanisms—such as whether nZnO upregulates genes involved in auxin biosynthesis or signaling—remain to be elucidated. Future transcriptomic or enzyme activity assays could directly test this hypothesis [63]. This nZnO-induced hormonal priming, along with other factors, could improve root growth and nutrient uptake, thereby collectively enhancing the plant’s overall tolerance under combined stress. This, in turn, has been shown to have a considerable impact on the IAA level, to promote root growth and water uptake, and to enhance the plant’s tolerance to heavy metals. Abdel et al. [64] found that nZnO could increase the potato’s GA_3_ content, thereby improving its salt tolerance. Furthermore, the application of nZnO has been demonstrated to enhance the level of ABA in plants, thereby increasing their tolerance to abiotic stress [65,66]. However, Cd treatment significantly reduced the ZT content in leaves, which is consistent with the findings of Procházková et al. [67]. Conversely, nZnO treatment increased the ZT content in lettuce leaves, which in turn promoted the de-epoxidation of the lutein cycle (DEPS) and attenuated the limitation of leaf photosynthesis by stomatal and non-stomatal factors [59].

In plants, oxidative stress is commonly associated with the accumulation of reactive oxygen species (ROS). The production of ROS has been demonstrated to contribute to the peroxidation of unsaturated fatty acids within the cell membrane, resulting in an augmentation of the MDA content [35].The generation of ROS can exert a positive effect on plant growth, albeit to a limited extent, while a further increase has been shown to exert a negative influence on plant growth [68]. In our study, the observed growth stimulation occurred concurrently with specific levels of ROS under Cd hormesis. This co-occurrence supports the possibility that ROS acted as signaling molecules, which can activate antioxidant defence and adaptive responses that are known to stimulate plant growth [69]. Furthermore, it has been demonstrated that ROS can function as signalling agents, capable of modulating physiological processes and thereby enhancing tolerance to abiotic stresses [70]. Our results indicate a nuanced role of ROS under Cd hormesis. The level of oxidative stress may not have been severely damaging and that ROS could potentially have acted as signaling molecules involved in the growth response [71]. This dual role of ROS as both a signaling molecule at low levels and a cytotoxic agent at high levels is consistent with the hormesis model reported in other leafy vegetables like pak choi (Brassica rapa) under cadmium stress [20]. As demonstrated in earlier studies, elevated levels of Cd have been observed to prompt a robust antioxidant defence response for a limited duration. Nevertheless, this induction is not enduring and is incapable of counteracting the Cd-induced oxidative burst [21]. Conversely, low Cd exposure can enhance the activities of antioxidant enzymes in some plants [72]. The ability of nZnO to enhance the antioxidant system aligns with findings in wheat, where ZnO nanoparticles upregulated the activity of SOD, POD, and CAT, which was correlated with reduced H₂O₂ and MDA levels under Cd stress [73]. Antioxidant enzymes play a crucial role in balancing the intracellular ROS level. SOD first converts O_2_^-^ to less toxic H_2_O_2_, after which APX, CAT and POD are responsible for reducing H_2_O_2_ to H_2_O to avoid membrane lipid peroxidation [74].Zinc has been identified as a component of SOD and copper/zinc isoenzymes, playing a crucial role in the stabilization of the zinc finger structure of proteins and the protection of membranes from oxidative damage [75].In addition, studies have shown that ZnO has the ability to increase the activity of antioxidant enzymes and enhance the expression of related genes in wheat [76], tomato [77], mung bean [34], and chili peppers [78]. These enhanced antioxidant functions help plants to scavenge more ROS generated by stress signals [79].

Our data demonstrate that cadmium stress induced a significant increase in the total phenolic content in lettuce. This finding is consistent with reports in other species such as mint [80]. and underscores a common plant defense response. The observed shift in Cd towards less mobile and toxic forms (e.g., F_R_ and F_HAC_) is likely facilitated, at least in part, by these elevated phenolic compounds, which are well-documented to chelate heavy metals into less bioavailable complexes, thereby reducing their translocation within the plant. While our correlation analysis does not establish a direct causal mechanism, the observed phenolic accumulation is a key component of the plant’s integrated defense strategy. This is because extensive previous literature has demonstrated that phenolics can act as metal chelators and antioxidants [81]. It is therefore plausible that the phenolics produced in response to Cd in our study contributed to reducing Cd mobility and toxicity through these well-documented mechanisms, although further molecular evidence would be required to confirm this specific pathway. Similarly, a study on mint reported a significant induction of phenolic compounds as a key defense mechanism against Cd-induced oxidative damage [80]. Foliar application of nZnO further increased the total phenolic content in lettuce tissues, exhibiting a clear dose-dependent effect. This phenomenon is in agreement with the findings of Abdellatif et al. [82]. We hypothesize that changes in nitrogen metabolism may explain for the effect on total phenol content. It has been demonstrated that plants are capable of altering nitrogen metabolism and thereby reducing primary metabolism when exposed to Cd. This, in turn, has been shown to reduce the precursors required for the synthesis of phenolic acids and flavonoid metabolites. Furthermore, it has been demonstrated that nZnO was able to increase nitrogen metabolism in the plant, which may also be the reason for the improvement in total phenol content [83].

Parrotta et al. hypothesised that the structure of the plant cell wall is a key factor in enhancing the tolerance of Cd-exposed plants, in which lignin is the most significant macromolecule in the cell wall [84]. It is generally accepted that a higher lignin content in the plant cell wall may enhance plant tolerance to Cd exposure. However, the protective effects of lignin are twofold. Firstly, lignin can reduce the permeability of the cell wall and provide Cd-binding sites, which are immobilized in the cell wall to protect the protoplasts. Conversely, overaccumulation of lignin in the root elongation zone, while it can curtail the uptake of Cd by the plant, will also impede the growth of the root system [85]. The induction of lignin metabolism under Cd stress is likely mediated by H_2_O_2_ signaling, which is known to promote the transcription of key genes (e.g., PAL, C4H) and enhance the activity of enzymes such as POD involved in monolignol polymerization [86,87]. Our findings, showing a coordinated upregulation of phenylpropanoid pathway enzyme activities (Fig 6), align with this established model. However, to confirm that this transcriptional regulation is operational in our system and is primed by nZnO, future work quantifying the expression of these key genes (e.g., PAL, 4CL, CAD) would be essential. Furthermore, the application of nZnO, particularly the high dose, further augmented this response. However, the overall growth data (Fig 2) indicate that the highest dose did not consistently yield the greatest biomass benefit, suggesting a complex dose-response relationship that may involve compensatory or stress-inducing effects at elevated concentrations. Together with the observed shift in Cd subcellular distribution towards the cell wall fraction (Fig 9), these data suggest that nZnO may mitigate Cd toxicity not only by enhancing antioxidant capacity but also by promoting lignin deposition, which is associated with reduced Cd translocation to edible shoots. As demonstrated in Fig 10, the application of nZnO has been shown to enhance the accumulation of lignin within plant material, with the majority of this accumulation occurring in the root system in close proximity to the above-ground portion of the plant. This suggests that nZnO-induced lignification did not reach levels that would inhibit root growth [88]. This nZnO-induced lignification in roots, which is associated with enhanced Cd sequestration in the apoplast, is consistent with a well-documented mechanism. For instance, research on Brassica species demonstrated that ZnO nanoparticles triggered cell wall remodeling and lignin deposition, which was directly linked to reduced Cd translocation to shoots [88]. In the present study, the treatment of plants with nZnO resulted in a significant increase in total phenolic content and the activities of PAL, CAD, C4H, and 4CL in comparison with Cd treatment. This effect was more pronounced at elevated levels of nZnO. Benakova et al. In addition, it was determined that low concentrations of nZnO were capable of increasing the lignin content of rape roots [89]. This effect may be linked to the upregulation of Zn metabolism genes, particularly those involved in the synthesis of key amino acids such as Phe, Cys, and Met [90]. Collectively, our findings indicate that nZnO enhances lignification, especially in roots, and alleviates Cd-induced oxidative stress.

Cadmium has been observed to compete with mineral nutrient ions for the same transport system, resulting in a deficiency of nutrients essential for plant growth and development. An investigation by Luyckx et al. [91] documented an adverse impact of Cd^2+^ on the absorption of plant nutrients. The effect of Cd on plant species is dependent on the species in question and the Cd concentration in the environment [92]. In the present study, Cd treatment resulted in a decrease in the concentrations of Cu, Fe, Mg and Zn in lettuce tissues, while concurrently increasing the level of Ca. As indicated by the findings of Matraszek et al., Lavres et al. [14] and Kumar et al. [93], analogous outcomes were obtained.It is hypothesised that the observed phenomenon may be attributable to a synergistic effect between Cd and Ca. This assertion is supported by the findings from the Cd treatment experiment, which revealed a substantial decrease in Zn content. This decline can be attributed not only to the competition between Cd and Zn for shared ion transporter proteins, but also to the increase in Ca content, which impedes Zn uptake by plants through their roots [94].In previous studies, it was reported that nZnO significantly ameliorated cadmium exposure in pea [95], corn [30], and wheat [73] due to the reduction of mineral elements. The observed restoration of Zn, Fe, and Mg levels by nZnO L in our study corroborates findings in rice, where foliar application of ZnO nanoparticles improved the uptake of these essential nutrients under Cd stress, likely by antagonizing Cd uptake and mitigating its phytotoxic effects on nutrient transporters [31]. In the present study, foliar spraying of nZnO significantly reduced Cd accumulation while differentially modulating nutrient homeostasis. The decrease in Cd content across treatments can be mechanistically explained by ionic competition and transporter regulation. As Zn and Cd share physicochemical similarities, the foliar-absorbed Zn likely downregulated the expression and activity of root metal transporters (e.g., ZIP and IRT families), which are non-selective and facilitate Cd^2+^ uptake [96]. This is supported by the observed dose-dependent increase in shoot Zn content, indicating systemic translocation of the applied nanoparticles or ions, which could exert this regulatory effect on root uptake mechanisms. Furthermore, the distinct nutrient profiles under low (nZnO L) and high (nZnO H) doses reveal a concentration-dependent interplay between nutrient acquisition and sequestration strategies. The nZnO L treatment enhanced the uptake of Zn, Fe, and Mg, suggesting a general alleviation of Cd-induced nutrient deprivation, potentially via the upregulation of non-specific metal transporters or improved root health. In contrast, the nZnO H treatment caused a significant decline in Ca content alongside the highest Zn accumulation. This suggests a strong ion antagonism between Zn^2+^ and Ca^2+^ at high concentration, possibly for apoplastic binding sites or shared influx channels, thereby inhibiting Ca uptake. The concomitant enhancement of root lignification In the present study, foliar spraying of nZnO effectively reduced Cd accumulation, but its impact on nutrient homeostasis was concentration-dependent. The low dose (50 μmol l^-1^ nZnO) mitigated Cd-induced nutrient deprivation, increasing the content of Zn, Fe, and Mg. However, the high dose (100 μmol l^-1^ nZnO) induced a new nutritional imbalance, characterized by Zn hyperaccumulation and a significant decline in Ca content. This phenomenon highlights a critical trade-off, where the efficacy of Cd mitigation must be weighed against potential phytotoxic effects at high nanoparticle concentrations. The decline in Ca uptake is likely due to ion antagonism between Zn^2+^ and Ca^2+^ for apoplastic binding sites and shared influx channels, an effect potentially exacerbated by the enhanced lignification of the root system [97], which may alter ion selectivity and mobility. While this study demonstrates the efficacy of foliar-applied nZnO, its potential environmental fate and ecological impacts cannot be overlooked, particularly for soil-based agriculture. A significant portion of foliar-applied nanoparticles can reach the soil through wash-off or leaf litter decomposition [98]. Once in the soil, nZnO can persist and potentially disrupt key ecosystem functions; for instance, studies have shown that nZnO can inhibit soil microbial activity, alter bacterial and fungal community structure, and reduce the activity of nutrient-cycling enzymes [99]. Therefore, while nZnO is a promising strategy for Cd mitigation, its application requires careful optimization of dosage not only to avoid phytotoxicity and nutritional imbalances, as observed herein, but also to mitigate potential broader ecological risks. The nutrient imbalances observed at high nZnO doses in this hydroponic study may signal broader ionic disruptions that could affect soil health and microbial processes. Plant roots primarily absorb Cd as Cd^2+^ ions or chlorinated complexes. Furthermore, environmental zinc levels have been demonstrated to exert a substantial influence on plant Cd levels, given that the majority of Cd is absorbed through zinc transport proteins such as *ZIP* and *IRT* [96].

The cell wall has been identified as the storage site for Cd, and it functions as the primary barrier and the main site of immobilization of Cd^2+^ from plant roots, thereby preventing Cd^2+^ from entering root protoplasts [100]. Once the Cd-binding capacity of the cell wall has been saturated, the sites of Cd accumulation in root cell protoplasts become primarily the cytoplasm and vesicles. Vesicles have been found to contain a significant quantity of sulfur-rich states and organic acids, which have been demonstrated to possess the ability to chelate and sequester Cd, thereby preventing damage to organelles caused by heavy metals [101]. As demonstrated in earlier research, plants have been found to sequester substantial quantities of Cd in the soluble fraction or within the cell wall, thereby mitigating the impact of Cd on organelles [102]. In the present study, it was observed that leaf spraying with nZnO increased the fraction of Cd in the cell wall and decreased the fraction of protoplasts and soluble fraction in comparison with Cd treatment, and the effect was more pronounced at higher concentrations. The application of leaf spray nZnO also led to a decrease in Cd content in subcellular fractions of lettuce roots. Cadmium in plants exists in a variety of chemical forms that differ in toxicity levels and transport capacity [103]. In general, the water-soluble inorganic F_W_ and the pectin-protein complexed form of FE are more readily transported into plant cells and are more toxic. The protein-bound form of F_NaCl_ is less toxic, although it interferes with normal enzyme functions. The insoluble forms of F_R_ and F_HAC_ are virtually non-toxic [103,104]. The findings of earlier research studies indicate that the presence of Cd in cabbage and autumn tomato is predominantly characterised by F_E_ within the pectin-protein complexation state. This observation is in alignment with the results obtained in the present study [105,106]. Foliar application of nZnO significantly reduced the F_E_ and F_NaCl_ fractions in the root system, while F_R_ and F_HAC_ were found to be significantly increased. This shift from more toxic and mobile forms (F_E_, F_W_) to less toxic and immobile forms (F_HAC_, F_R_) is a critical mechanism by which amendments mitigate Cd toxicity. A parallel study on Sedum alfredii demonstrated that zinc application facilitated the conversion of Cd into insoluble phosphate complexes (F_HAC_) and oxalates (F_R_), thereby reducing its bioavailability and toxicity [51]. The results of the present study indicate that nZnO may increase the lignin, pectin and protein contents in roots, alter the chemical form of Cd in the root system and stabilise it in the cell wall, and impede the uptake of Cd in lettuce roots and its translocation to the leaves [90,107,108].

Finally, the overall phytotoxicity of foliar nZnO treatment was assessed against the hormesis effects of Cd using Pearson correlation analysis (Fig 10), a normalized heat map (Fig 11), and principal component analysis (Fig 12) of the target metabolites in lettuce. The results demonstrated that the foliar application of nZnO was associated with a reduction in the concentration of Cd in the leaves and roots of lettuce, which showed a significant negative correlation with the lignin content. This correlation was proportional to the concentration of foliar nZnO.

Based on our findings, we propose a unified model for nZnO-mediated mitigation of Cd toxicity in lettuce, which integrates hormonal, biochemical, and physiological responses. The nZnO-induced hormonal shifts, particularly the potential rise in IAA and ABA, likely act as a primary event. This hormonal priming orchestrates the downstream biochemical responses; ABA and an initial, non-damaging ROS burst can upregulate the phenylpropanoid pathway, simultaneously enhancing the synthesis of protective phenolic compounds for metal chelation and antioxidation, and lignin for apoplastic sequestration. Furthermore, the applied zinc itself is a fundamental component of this integrated system. It acts as a cofactor for key enzymes like superoxide dismutase in the antioxidant system and competes with Cd for uptake at transport sites, directly reducing influx. The improvement in nutrient homeostasis supports the activity of metal-dependent enzymes, while the enhanced lignification of the root apoplast provides a physical barrier that functionally complements the reduced uptake by immobilizing any Cd that enters. In conclusion, our findings suggest that nZnO may act as a multi-faceted priming agent that enhances the plant’s hormonal signaling, antioxidant capacity, metal chelation potential, and physical barrier formation. The integration of these responses correlates with reduced Cd mobility and toxicity in lettuce. However, this study was conducted under controlled greenhouse conditions over a relatively short term. Before any potential agricultural application, the environmental risks of nZnO, including its long-term stability in soil and effects on ecosystem health, must be thoroughly evaluated through dedicated ecotoxicological studies and field trials.

### Limitations and future perspectives

In addition to the discussed limitations of the hydroponic system and exposure duration, our study primarily provides physiological and biochemical insights. The proposed models for hormone-mediated growth promotion and H₂O₂-triggered lignification, while consistent with our data and literature, require direct molecular validation. Future research should therefore employ transcriptomic and proteomic approaches to quantify the expression of key genes related to phytohormone biosynthesis (e.g., YUCCA, GA20ox), metal transport (e.g., *ZIP*, *IRT*), and the phenylpropanoid pathway (e.g., PAL, C4H, CAD). Such work would solidify the mechanistic links between nZnO application, hormonal changes, enhanced lignification, and reduced Cd uptake and translocation.

Based on these limitations, future research should focus on: (1) validating the efficacy of foliar nZnO application in long-term field trials using Cd-contaminated agricultural soils, with particular attention to the environmental fate of nZnO, including its persistence, transformation (e.g., dissolution and sulfidation), and potential leaching, and how these processes subsequently influence Cd speciation and bioavailability [109,110]; (2) employing transcriptomic and proteomic approaches to identify key genes and proteins regulated by nZnO under Cd hormesis, which would provide direct molecular evidence for the observed hormonal and lignin metabolism changes; and (3) conducting comprehensive ecotoxicological assessments to evaluate the potential accumulation of nZnO in the environment and its impact on soil microbial communities. Addressing these points will be crucial for translating this promising strategy into safe and effective agricultural practice.

## Conclusions

Our study demonstrates that lettuce exhibits a pronounced hormetic response to low-dose Cd (2.5 μmol L⁻¹) in hydroponics, whereby growth is stimulated despite the element’s inherent toxicity. We found that foliar spraying of nZnO is a feasible strategy to tackle the unique challenge of plants thriving but becoming unsafe for consumption. The primary benefits of nZnO included a significant reduction in cadmium accumulation within edible leaves and enhanced detoxification. These effects appear to be mediated through multiple mechanisms: nZnO application modulated endogenous hormones and ROS homeostasis, which in turn was associated with boosted lignin biosynthesis. The subsequent lignification of the root apoplast provided more binding sites and a stronger physical barrier, which enhanced the sequestration of Cd in the cell wall and reduced its translocation to shoots, as supported by the subcellular distribution analysis. Furthermore, this process contributed to immobilizing Cd and reducing the proportion of its mobile and toxic chemical forms in the roots.

However, a balanced assessment must also consider potential drawbacks. Our results indicate that a high concentration of nZnO can disturb plant nutrient homeostasis. Furthermore, as discussed, the potential for soil accumulation and negative impacts on soil biota presents a significant environmental consideration that extends beyond direct phytotoxicity. Therefore, although foliar nZnO application is a promising strategy for enhancing the safety of vegetables grown in mildly contaminated soils, the dosage requires careful optimization. We strongly recommend that future work progresses to long-term field trials under realistic agricultural conditions. These trials must integrate dedicated ecotoxicological assessments to monitor the soil fate of nZnO and its impacts on microbial communities and ecosystem functions, ensuring a comprehensive safety profile before any large-scale agricultural application is considered.

## Supporting information

S1 Fig (PDF)

S2 Fig (PDF)

S3 Fig (PDF)

S4 Fig (PDF)

S5 Fig (PDF)

S6 Fig (PDF)

S7 Fig (PDF)

S8 Fig (PDF)

S9 Fig (PDF)

S10 Fig (PDF)

S11 Fig (PDF)

S12 Fig (PDF)
