# Supplementary material for: Effect of zinc oxide nanoparticles (nZnO) on antioxidant defense, lignin metabolism and cadmium subcellular distribution in lettuce (Lactuca sativa L) under low-dose cadmium stress (hormesis)
Source: PLoS One. 2025 Dec 4;20(12):e0337953. doi: 10.1371/journal.pone.0337953 (PMC12677453; doi:10.1371/journal.pone.0337953)
Supplement: S1 Fig — (PDF) [file pone.0337953.s001.pdf]

Supporting information

S1_file Fig 1 A、B

Treatment	Leaf FW	Root FW	Leaf DW	Root DW
CK	30.35	3.25	1.52	0.2
CK	30.56	3.37	1.53	0.22
CK	32.19	3.48	1.61	0.18
CK	30.07	3.43	1.59	0.21
CK	32.88	3.38	1.64	0.21
Cd 1	31.66	3.54	1.68	0.24
Cd 1	32.07	3.52	1.60	0.22
Cd 1	31.25	3.47	1.63	0.21
Cd 1	34.37	3.51	1.74	0.25
Cd 1	31.39	3.6	1.72	0.26
Cd 2.5	34.03	3.87	1.7	0.27
Cd 2.5	37.43	3.56	1.77	0.21
Cd 2.5	31.65	3.79	1.68	0.27
Cd 2.5	35.5	3.96	1.72	0.27
Cd 2.5	35.21	3.94	1.78	0.29
Cd 5	25.05	2.53	1.25	0.15
Cd 5	25.76	2.42	1.29	0.15
Cd 5	24.78	2.63	1.24	0.16
Cd 5	24.85	4.18	1.75	0.28
Cd 5	26.38	4.06	1.76	0.28
Cd 10	15.18	1.52	0.76	0.09
Cd 10	15.94	1.37	0.8	0.13
Cd 10	16.44	1.48	0.82	0.12
Cd 10	16.55	1.61	0.81	0.1
Cd 10	16.27	1.57	0.76	0.09

S1_file Fig 1 C

Treatment	Leaf	Root
Cd 1	1.50	4.05
Cd 1	1.44	4.09
Cd 1	1.53	3.89
Cd 2.5	4.42	13.84
Cd 2.5	4.78	15.50
Cd 2.5	4.16	13.61
Cd 5	7.57	23.11
Cd 5	7.51	22.82
Cd 5	7.46	24.64
Cd 10	10.10	37.30
Cd 10	10.82	38.07
Cd 10	11.89	37.15
