# Supplementary material for: Effect of zinc oxide nanoparticles (nZnO) on antioxidant defense, lignin metabolism and cadmium subcellular distribution in lettuce (Lactuca sativa L) under low-dose cadmium stress (hormesis)
Source: PLoS One. 2025 Dec 4;20(12):e0337953. doi: 10.1371/journal.pone.0337953 (PMC12677453; doi:10.1371/journal.pone.0337953)
Supplement: S2 Fig — (PDF) [file pone.0337953.s002.pdf]

S2_file Fig 2

Treatment	Leaf FW	Leaf DW	Root FW	Root DW
CK	31.35	1.53	3.45	0.21
CK	31.56	1.61	3.13	0.2
CK	30.19	1.51	3.28	0.23
CK	31.16	1.6	3.57	0.23
CK	32.22	1.49	2.88	0.21
Cd	33.03	1.69	3.56	0.29
Cd	35.43	1.54	3.53	0.28
Cd	34.65	1.86	3.55	0.28
Cd	34.85	1.71	3.5	0.29
Cd	32.88	1.48	3.33	0.28
nZnO L	38.6	1.82	4.22	0.37
nZnO L	37.83	1.77	4.09	0.34
nZnO L	35.9	1.93	4.18	0.33
nZnO L	39.99	1.65	4.61	0.36
nZnO L	37.68	1.9	3.89	0.34
nZnO H	35.45	1.77	4.48	0.35
nZnO H	34.74	1.75	4.39	0.33
nZnO H	32.61	1.88	4.17	0.36
nZnO H	37.54	1.67	4.85	0.36
nZnO H	34.11	1.86	4.37	0.31
