# Supplementary material for: Effect of zinc oxide nanoparticles (nZnO) on antioxidant defense, lignin metabolism and cadmium subcellular distribution in lettuce (Lactuca sativa L) under low-dose cadmium stress (hormesis)
Source: PLoS One. 2025 Dec 4;20(12):e0337953. doi: 10.1371/journal.pone.0337953 (PMC12677453; doi:10.1371/journal.pone.0337953)
Supplement: S3 Fig — (PDF) [file pone.0337953.s003.pdf]

S3_file Fig 3

Leaf	GA3	ZT	IAA	ABA
CK	38.31	36.50	36.57	79.27
CK	38.48	37.23	34.22	86.40
CK	39.53	39.42	36.57	73.72
Cd	43.61	34.91	40.16	88.75
Cd	43.07	33.17	37.35	95.85
Cd	43.53	33.52	36.14	96.74
nZnO L	47.51	41.76	45.73	106.01
nZnO L	46.04	38.42	45.56	95.41
nZnO L	47.33	42.17	43.53	102.83
nZnO H	42.93	45.87	49.31	129.83
nZnO H	47.22	46.79	46.77	126.85
nZnO H	41.64	47.25	48.98	132.43
Root				
CK	9.52	4.16	13.52	6.72
CK	8.69	4.64	13.16	7.01
CK	8.80	4.83	14.08	6.49
Cd	11.13	4.88	13.38	7.48
Cd	10.05	4.86	15.02	8.02
Cd	9.64	4.74	14.88	7.40
nZnO L	12.32	4.72	15.14	10.73
nZnO L	13.21	5.10	15.79	9.52
nZnO L	12.34	4.44	15.73	9.59
nZnO H	13.09	4.74	15.28	11.63
nZnO H	12.60	4.79	15.17	11.47
nZnO H	12.87	4.51	15.38	11.41
