# Supplementary material for: Effect of zinc oxide nanoparticles (nZnO) on antioxidant defense, lignin metabolism and cadmium subcellular distribution in lettuce (Lactuca sativa L) under low-dose cadmium stress (hormesis)
Source: PLoS One. 2025 Dec 4;20(12):e0337953. doi: 10.1371/journal.pone.0337953 (PMC12677453; doi:10.1371/journal.pone.0337953)
Supplement: S4 Fig — (PDF) [file pone.0337953.s004.pdf]

S4_file Fig 4

Leaf	MDA	H_2O_2	O_2^-
CK	2.11	1.80	1.38
CK	2.05	1.70	1.32
CK	2.27	1.90	1.51
Cd	3.17	2.71	2.05
Cd	3.00	2.42	1.94
Cd	3.17	2.53	2.08
nZnO L	2.62	2.05	1.69
nZnO L	2.51	1.96	1.72
nZnO L	2.61	1.90	1.93
nZnO H	2.57	2.06	1.82
nZnO H	2.76	2.20	1.92
nZnO H	2.53	2.12	1.71
Root			
CK	3.17	2.70	2.11
CK	3.25	2.55	2.23
CK	3.46	2.85	1.75
Cd	4.76	4.65	3.75
Cd	4.50	4.23	3.56
Cd	4.76	4.88	3.44
nZnO L	3.93	4.36	3.00
nZnO L	4.08	4.19	3.03
nZnO L	4.16	4.30	3.21
nZnO H	4.39	4.45	3.28
nZnO H	4.31	4.38	3.19
nZnO H	4.25	4.47	3.27
