# Supplementary material for: Effect of zinc oxide nanoparticles (nZnO) on antioxidant defense, lignin metabolism and cadmium subcellular distribution in lettuce (Lactuca sativa L) under low-dose cadmium stress (hormesis)
Source: PLoS One. 2025 Dec 4;20(12):e0337953. doi: 10.1371/journal.pone.0337953 (PMC12677453; doi:10.1371/journal.pone.0337953)
Supplement: S5 Fig — (PDF) [file pone.0337953.s005.pdf]

S5_file Fig 5

Leaf	SOD	POD	CAT	APX
CK	59.46	1.02	4.65	27.98
CK	64.30	0.98	4.75	24.98
CK	62.27	1.12	4.30	26.42
Cd	74.83	1.71	6.36	29.84
Cd	78.59	1.65	6.88	31.77
Cd	75.10	1.75	7.07	30.91
nZnO L	79.69	1.84	7.38	35.25
nZnO L	83.43	1.91	7.53	34.01
nZnO L	78.91	1.85	7.62	32.14
nZnO H	89.29	2.06	8.96	36.52
nZnO H	84.90	2.14	8.70	37.61
nZnO H	87.04	2.17	7.92	39.46
Root				
CK	97.84	3.32	4.05	13.99
CK	100.29	3.46	4.15	12.49
CK	95.68	3.56	4.30	13.21
Cd	112.45	4.35	4.88	15.92
Cd	104.58	4.22	4.92	15.89
Cd	102.33	4.04	4.61	15.96
nZnO L	121.95	4.99	4.93	16.13
nZnO L	119.03	5.07	5.17	17.01
nZnO L	123.67	5.12	5.46	18.07
nZnO H	131.79	5.86	5.40	18.26
nZnO H	130.47	5.37	5.29	18.80
nZnO H	141.01	5.88	5.77	17.73
