# Supplementary material for: Effect of zinc oxide nanoparticles (nZnO) on antioxidant defense, lignin metabolism and cadmium subcellular distribution in lettuce (Lactuca sativa L) under low-dose cadmium stress (hormesis)
Source: PLoS One. 2025 Dec 4;20(12):e0337953. doi: 10.1371/journal.pone.0337953 (PMC12677453; doi:10.1371/journal.pone.0337953)
Supplement: S6 Fig — (PDF) [file pone.0337953.s006.pdf]

S6_file Fig 6

Leaf	PAL	C4H	4CL	CAD	Total phenols	Lignin OD 280 g^{-1}
CK	13.55	4.22	3.75	6.81	920.42	0.33
CK	13.36	4.46	3.68	5.72	873.05	0.30
CK	13.77	4.03	3.85	6.87	873.44	0.29
Cd	14.86	5.14	4.44	8.48	904.97	0.44
Cd	15.18	5.21	4.40	8.06	964.91	0.46
Cd	14.12	5.19	4.86	8.95	955.84	0.47
nZnO L	15.86	6.20	5.36	9.30	1129.84	0.54
nZnO L	16.44	6.16	6.03	10.79	1155.94	0.60
nZnO L	15.93	5.82	5.87	10.54	1087.80	0.61
nZnO H	18.09	6.74	6.60	11.94	1445.00	0.67
nZnO H	17.85	7.15	6.05	12.59	1347.69	0.68
nZnO H	18.89	6.98	6.39	11.10	1334.44	0.72
Root						
CK	5.57	2.05	2.12	1.97	199.26	0.39
CK	5.14	2.25	2.76	2.30	212.99	0.38
CK	5.28	1.96	2.86	2.19	208.16	0.40
Cd	6.04	2.80	3.97	3.31	224.26	0.70
Cd	5.74	3.02	3.38	2.82	238.51	0.62
Cd	6.13	2.64	4.23	3.53	226.37	0.66
nZnO L	6.70	3.68	4.21	3.51	234.44	0.93
nZnO L	7.30	3.70	4.55	3.79	244.26	1.06
nZnO L	6.28	3.57	4.36	3.64	247.00	1.15
nZnO H	7.65	3.98	4.83	4.19	269.06	1.47
nZnO H	8.22	4.03	4.51	3.93	280.40	1.49
nZnO H	8.13	4.16	4.52	4.29	268.58	1.51
