# Supplementary material for: Effect of zinc oxide nanoparticles (nZnO) on antioxidant defense, lignin metabolism and cadmium subcellular distribution in lettuce (Lactuca sativa L) under low-dose cadmium stress (hormesis)
Source: PLoS One. 2025 Dec 4;20(12):e0337953. doi: 10.1371/journal.pone.0337953 (PMC12677453; doi:10.1371/journal.pone.0337953)
Supplement: S7 Fig — (PDF) [file pone.0337953.s007.pdf]

S7_file Fig 7

Leaf	Cu	Fe	Zn	Mg	Ca
CK	0.79	0.75	0.10	1.00	0.87
CK	1.00	0.83	0.11	0.98	0.00
CK	0.80	0.94	0.06	0.98	0.25
Cd	0.41	0.00	0.02	0.47	1.00
Cd	0.46	0.19	0.00	0.19	0.73
Cd	0.40	0.16	0.02	0.23	0.96
nZnO L	0.39	0.79	0.68	0.71	0.80
nZnO L	0.26	1.00	0.75	0.78	0.51
nZnO L	0.33	0.92	0.69	0.72	0.69
nZnO H	0.06	0.35	0.90	0.14	0.30
nZnO H	0.00	0.30	1.00	0.00	0.32
nZnO H	0.19	0.30	0.96	0.28	0.03
Root					
CK	1.00	1.00	0.31	1.00	0.02
CK	0.94	0.54	0.42	0.79	0.00
CK	1.00	0.94	0.22	0.84	0.00
Cd	0.54	0.00	0.14	0.45	0.37
Cd	0.44	0.19	0.05	0.09	0.40
Cd	0.50	0.15	0.00	0.38	0.49
nZnO L	0.32	0.53	0.57	0.65	0.72
nZnO L	0.52	0.98	0.50	0.62	1.00
nZnO L	0.44	0.83	0.65	0.41	0.79
nZnO H	0.06	0.34	0.95	0.14	0.00
nZnO H	0.20	0.20	0.83	0.00	0.10
nZnO H	0.00	0.10	1.00	0.02	0.13
