# Supplementary material for: Effect of zinc oxide nanoparticles (nZnO) on antioxidant defense, lignin metabolism and cadmium subcellular distribution in lettuce (Lactuca sativa L) under low-dose cadmium stress (hormesis)
Source: PLoS One. 2025 Dec 4;20(12):e0337953. doi: 10.1371/journal.pone.0337953 (PMC12677453; doi:10.1371/journal.pone.0337953)
Supplement: S8 Fig — (PDF) [file pone.0337953.s008.pdf]

S8_file Fig 8

Treatment	Leaf	Root	TF
Cd	4.26	14.47	0.29
Cd	4.81	14.69	0.33
Cd	4.58	14.95	0.31
nZnO L	2.81	12.94	0.22
nZnO L	2.87	13.64	0.21
nZnO L	2.97	13.20	0.22
nZnO H	1.50	11.35	0.12
nZnO H	1.56	11.06	0.15
nZnO H	1.54	11.32	0.14
