# Supplementary material for: Effect of zinc oxide nanoparticles (nZnO) on antioxidant defense, lignin metabolism and cadmium subcellular distribution in lettuce (Lactuca sativa L) under low-dose cadmium stress (hormesis)
Source: PLoS One. 2025 Dec 4;20(12):e0337953. doi: 10.1371/journal.pone.0337953 (PMC12677453; doi:10.1371/journal.pone.0337953)
Supplement: S9 Fig — (PDF) [file pone.0337953.s009.pdf]

S9_file Fig 9 A

Root	FI	FII	FIII
Cd	34.06%	8.47%	57.41%
Cd	35.72%	8.68%	55.62%
Cd	34.82%	8.27%	56.82%
nZnO L	41.89%	9.98%	48.07%
nZnO L	41.31%	10.18%	48.59%
nZnO L	42.39%	10.50%	46.96%
nZnO H	47.27%	12.44%	40.53%
nZnO H	48.73%	11.79%	39.45%
nZnO H	48.81%	11.48%	39.74%

S9_file Fig 9 B

Root	FE	FW	FNaCl	FHAC	FHCl	FR
Cd	25.43%	22.44%	29.07%	18.82%	3.46%	0.75%
Cd	24.94%	22.12%	29.94%	19.38%	3.29%	0.73%
Cd	24.41%	23.77%	27.90%	19.88%	3.32%	0.75%
nZnO L	20.44%	17.48%	33.90%	22.05%	4.55%	1.82%
nZnO L	20.85%	18.00%	32.58%	21.85%	4.69%	1.91%
nZnO L	20.03%	18.18%	33.06%	22.39%	4.60%	1.86%
nZnO H	17.53%	15.55%	35.17%	24.37%	5.50%	1.84%
nZnO H	16.39%	16.49%	35.52%	24.14%	5.48%	1.83%
nZnO H	16.67%	16.08%	36.28%	23.71%	5.48%	1.84%
