# Supplementary material for: Effect of zinc oxide nanoparticles (nZnO) on antioxidant defense, lignin metabolism and cadmium subcellular distribution in lettuce (Lactuca sativa L) under low-dose cadmium stress (hormesis)
Source: PLoS One. 2025 Dec 4;20(12):e0337953. doi: 10.1371/journal.pone.0337953 (PMC12677453; doi:10.1371/journal.pone.0337953)
Supplement: S10 Fig — (PDF) [file pone.0337953.s010.pdf]

S10_file Fig 10 A、B

Label	Leaf FW	Leaf DW	GA3	ZT	IAA
Leaf FW	1.00	0.73	1.00	0.22	0.37
Leaf DW	0.73	1.00	0.71	0.83	0.90
GA_3	1.00	0.71	1.00	0.18	0.33
ZT	0.22	0.83	0.18	1.00	0.99
IAA	0.37	0.90	0.33	0.99	1.00
ABA	-0.14	0.57	-0.18	0.93	0.87
MDA	-0.70	-1.00	-0.66	-0.86	-0.92
H_2O_2	-0.83	-0.99	-0.80	-0.73	-0.83
O_2^-	-0.76	-1.00	-0.73	-0.81	-0.89
SOD	-0.48	-0.95	-0.45	-0.96	-0.99
POD	0.07	0.73	0.03	0.99	0.95
CAT	-0.79	-1.00	-0.76	-0.78	-0.86
APX	-0.41	-0.92	-0.38	-0.98	-1.00
PAL	0.04	0.71	0.00	0.98	0.94
C4H	0.17	0.80	0.13	1.00	0.98
4CL	0.36	0.90	0.32	0.99	1.00
CAD	0.18	0.80	0.14	1.00	0.98
Total phenols	0.09	0.74	0.05	0.99	0.96
Lignin	0.27	0.85	0.23	1.00	0.99
Leaf Cd	-0.24	-0.83	-0.20	-1.00	-0.99
	ABA	MDA	H2O2	O2-	SOD
Leaf FW	-0.14	-0.70	-0.83	-0.76	-0.48
Leaf DW	0.57	-1.00	-0.99	-1.00	-0.95
GA_3	-0.18	-0.66	-0.80	-0.73	-0.45
ZT	0.93	-0.86	-0.73	-0.81	-0.96
IAA	0.87	-0.92	-0.83	-0.89	-0.99
ABA	1.00	-0.61	-0.44	-0.54	-0.80
MDA	-0.61	1.00	0.98	1.00	0.97
H_2O_2	-0.44	0.98	1.00	0.99	0.89
O_2^-	-0.54	1.00	0.99	1.00	0.94
SOD	-0.80	0.97	0.89	0.94	1.00
POD	0.98	-0.76	-0.62	-0.70	-0.91
CAT	-0.50	0.99	1.00	1.00	0.92
APX	-0.84	0.94	0.86	0.91	1.00
PAL	0.98	-0.75	-0.60	-0.69	-0.89
C4H	0.95	-0.83	-0.70	-0.78	-0.95
4CL	0.87	-0.92	-0.82	-0.88	-0.99
CAD	0.95	-0.83	-0.71	-0.78	-0.95
Total phenols	0.97	-0.78	-0.63	-0.72	-0.91
Lignin	0.91	-0.88	-0.77	-0.83	-0.97
Leaf Cd	-0.93	0.86	0.74	0.81	0.96

	POD	CAT	APX	PAL	C4H
Leaf FW	0.07	-0.79	-0.41	0.04	0.17
Leaf DW	0.73	-1.00	-0.92	0.71	0.80
GA_3	0.03	-0.76	-0.38	0.00	0.13
ZT	0.99	-0.78	-0.98	0.98	1.00
IAA	0.95	-0.86	-1.00	0.94	0.98
ABA	0.98	-0.50	-0.84	0.98	0.95
MDA	-0.76	0.99	0.94	-0.75	-0.83
H_2O_2	-0.62	1.00	0.86	-0.60	-0.70
O_2^-	-0.70	1.00	0.91	-0.69	-0.78
SOD	-0.91	0.92	1.00	-0.89	-0.95
POD	1.00	-0.67	-0.94	1.00	0.99
CAT	-0.67	1.00	0.89	-0.65	-0.74
APX	-0.94	0.89	1.00	-0.93	-0.97
PAL	1.00	-0.65	-0.93	1.00	0.99
C4H	0.99	-0.74	-0.97	0.99	1.00
4CL	0.96	-0.86	-1.00	0.95	0.98
CAD	0.99	-0.75	-0.97	0.99	1.00
Total phenols	1.00	-0.68	-0.94	1.00	1.00
Lignin	0.98	-0.81	-0.99	0.97	1.00
Leaf Cd	-0.99	0.78	0.98	-0.98	-1.00
	4CL	CAD	Total phenols	Lignin	Leaf Cd
Leaf FW	0.36	0.18	0.09	0.27	-0.24
Leaf DW	0.90	0.80	0.74	0.85	-0.83
GA_3	0.32	0.14	0.05	0.23	-0.20
ZT	0.99	1.00	0.99	1.00	-1.00
IAA	1.00	0.98	0.96	0.99	-0.99
ABA	0.87	0.95	0.97	0.91	-0.93
MDA	-0.92	-0.83	-0.78	-0.88	0.86
H_2O_2	-0.82	-0.71	-0.63	-0.77	0.74
O_2^-	-0.88	-0.78	-0.72	-0.83	0.81
SOD	-0.99	-0.95	-0.91	-0.97	0.96
POD	0.96	0.99	1.00	0.98	-0.99
CAT	-0.86	-0.75	-0.68	-0.81	0.78
APX	-1.00	-0.97	-0.94	-0.99	0.98
PAL	0.95	0.99	1.00	0.97	-0.98
C4H	0.98	1.00	1.00	1.00	-1.00
4CL	1.00	0.98	0.96	1.00	-0.99
CAD	0.98	1.00	1.00	1.00	-1.00
Total phenols	0.96	1.00	1.00	0.98	-0.99
Lignin	1.00	1.00	0.98	1.00	-1.00
Leaf Cd	-0.99	-1.00	-0.99	-1.00	1.00

S10_file Fig 10 C、D

Label	Root FW	Root DW	GA3	ZT	IAA
Root FW	1.00	0.93	0.99	-0.95	0.88
Root DW	0.93	1.00	0.98	-0.77	0.99
GA_3	0.99	0.98	1.00	-0.89	0.95
ZT	-0.95	-0.77	-0.89	1.00	-0.70
IAA	0.88	0.99	0.95	-0.70	1.00
ABA	0.99	0.86	0.95	-0.99	0.80
MDA	-0.78	-0.96	-0.88	0.56	-0.98
H_2O_2	-0.73	-0.93	-0.83	0.48	-0.96
O_2^-	-0.83	-0.98	-0.91	0.62	-0.99
SOD	-0.68	-0.91	-0.80	0.43	-0.94
POD	0.98	0.84	0.94	-0.99	0.78
CAT	-0.66	-0.89	-0.78	0.40	-0.93
APX	-0.99	-0.96	-1.00	0.92	-0.93
PAL	0.92	0.71	0.84	-1.00	0.63
C4H	1.00	0.89	0.97	-0.97	0.85
4CL	1.00	0.90	0.97	-0.97	0.85
CAD	0.95	0.77	0.89	-1.00	0.70
Total phenols	0.87	0.62	0.77	-0.98	0.54
Lignin	0.95	0.76	0.88	-1.00	0.69
Root Cd	-0.93	-0.73	-0.86	1.00	-0.66
	ABA	MDA	H2O2	O2-	SOD
Root FW	0.99	-0.78	-0.73	-0.83	-0.68
Root DW	0.86	-0.96	-0.93	-0.98	-0.91
GA_3	0.95	-0.88	-0.83	-0.91	-0.80
ZT	-0.99	0.56	0.48	0.62	0.43
IAA	0.80	-0.98	-0.96	-0.99	-0.94
ABA	1.00	-0.67	-0.61	-0.73	-0.56
MDA	-0.67	1.00	1.00	1.00	0.99
H_2O_2	-0.61	1.00	1.00	0.99	1.00
O_2^-	-0.73	1.00	0.99	1.00	0.97
SOD	-0.56	0.99	1.00	0.97	1.00
POD	1.00	-0.65	-0.59	-0.71	-0.53
CAT	-0.53	0.98	1.00	0.97	1.00
APX	-0.97	0.84	0.79	0.88	0.75
PAL	0.97	-0.48	-0.40	-0.55	-0.34
C4H	1.00	-0.73	-0.67	-0.78	-0.62
4CL	1.00	-0.74	-0.68	-0.79	-0.63
CAD	0.99	-0.56	-0.48	-0.62	-0.43
Total phenols	0.94	-0.37	-0.29	-0.45	-0.23
Lignin	0.99	-0.55	-0.47	-0.61	-0.42
Root Cd	-0.98	0.51	0.43	0.57	0.37
	POD	CAT	APX	PAL	C4H

Root FW	0.98	-0.66	-0.99	0.92	1.00
Root DW	0.84	-0.89	-0.96	0.71	0.89
GA_3	0.94	-0.78	-1.00	0.84	0.97
ZT	-0.99	0.40	0.92	-1.00	-0.97
IAA	0.78	-0.93	-0.93	0.63	0.85
ABA	1.00	-0.53	-0.97	0.97	1.00
MDA	-0.65	0.98	0.84	-0.48	-0.73
H_2O_2	-0.59	1.00	0.79	-0.40	-0.67
O_2^-	-0.71	0.97	0.88	-0.55	-0.78
SOD	-0.53	1.00	0.75	-0.34	-0.62
POD	1.00	-0.51	-0.96	0.98	0.99
CAT	-0.51	1.00	0.73	-0.32	-0.60
APX	-0.96	0.73	1.00	-0.88	-0.98
PAL	0.98	-0.32	-0.88	1.00	0.95
C4H	0.99	-0.60	-0.98	0.95	1.00
4CL	0.99	-0.61	-0.99	0.94	1.00
CAD	0.99	-0.40	-0.92	1.00	0.97
Total phenols	0.95	-0.20	-0.81	0.99	0.91
Lignin	0.99	-0.39	-0.91	1.00	0.97
Root Cd	-0.98	0.35	0.89	-1.00	-0.96
	4CL	CAD	Total phenols	Lignin	Root Cd
Root FW	1.00	0.95	0.87	0.95	-0.93
Root DW	0.90	0.77	0.62	0.76	-0.73
GA_3	0.97	0.89	0.77	0.88	-0.86
ZT	-0.97	-1.00	-0.98	-1.00	1.00
IAA	0.85	0.70	0.54	0.69	-0.66
ABA	1.00	0.99	0.94	0.99	-0.98
MDA	-0.74	-0.56	-0.37	-0.55	0.51
H_2O_2	-0.68	-0.48	-0.29	-0.47	0.43
O_2^-	-0.79	-0.62	-0.45	-0.61	0.57
SOD	-0.63	-0.43	-0.23	-0.42	0.37
POD	0.99	0.99	0.95	0.99	-0.98
CAT	-0.61	-0.40	-0.20	-0.39	0.35
APX	-0.99	-0.92	-0.81	-0.91	0.89
PAL	0.94	1.00	0.99	1.00	-1.00
C4H	1.00	0.97	0.91	0.97	-0.96
4CL	1.00	0.97	0.90	0.97	-0.95
CAD	0.97	1.00	0.98	1.00	-1.00
Total phenols	0.90	0.98	1.00	0.98	-0.99
Lignin	0.97	1.00	0.98	1.00	-1.00
Root Cd	-0.95	-1.00	-0.99	-1.00	1.00
