# Supplementary material for: Effect of zinc oxide nanoparticles (nZnO) on antioxidant defense, lignin metabolism and cadmium subcellular distribution in lettuce (Lactuca sativa L) under low-dose cadmium stress (hormesis)
Source: PLoS One. 2025 Dec 4;20(12):e0337953. doi: 10.1371/journal.pone.0337953 (PMC12677453; doi:10.1371/journal.pone.0337953)
Supplement: S11 Fig — (PDF) [file pone.0337953.s011.pdf]

S11_file Fig 11

Treatment	L-CK	L-Cd	L-nZnO L	L-nZnO H
FW	-1.20	-0.15	1.24	0.11
DW	-1.04	-0.31	0.77	0.58
GA_3	-1.35	0.04	1.11	0.20
ZT	-0.41	-1.17	0.21	1.37
IAA	-1.08	-0.70	0.58	1.20
ABA	-1.10	-0.38	0.01	1.47
MDA	-1.28	1.35	-0.09	0.02
H_2O_2	-1.03	1.45	-0.47	0.05
O_2^-	-1.41	1.07	0.10	0.24
SOD	-1.33	1.31	0.16	-0.14
POD	-1.53	0.05	0.44	1.05
CAT	-1.44	1.17	0.01	0.26
APX	-1.25	1.33	0.18	-0.26
PAL	-1.12	-0.50	0.22	1.40
C4H	-1.29	-0.40	0.42	1.26
4CL	-1.25	-0.50	0.60	1.15
CAD	-1.29	-0.35	0.44	1.20
Total phenols	-0.96	-0.99	-0.83	-0.70
Lignin	-1.34	-1.10	-0.74	-0.35
Cd	0.00	1.19	-0.08	-1.11
	R-CK	R-Cd	R-nZnO L	R-nZnO H
FW	-1.08	-0.66	0.64	1.10
DW	-1.44	-0.24	0.89	0.79
GA_3	-1.25	-0.52	0.82	0.95
ZT	-0.65	0.52	0.22	-0.09
IAA	-1.21	-0.31	0.91	0.61
ABA	-1.11	-0.66	0.50	1.28
MDA	-1.47	1.09	-0.05	0.43
H_2O_2	-1.61	0.72	0.35	0.54
O_2^-	-1.54	0.96	0.15	0.42
SOD	-0.92	1.33	-0.78	0.36
POD	-1.28	-0.44	0.50	1.22
CAT	-1.36	1.06	-0.20	0.51
APX	-1.33	1.29	0.13	-0.09
PAL	-1.10	-0.51	0.23	1.38
C4H	-1.33	-0.42	0.62	1.13
4CL	-1.47	0.00	0.59	0.88
CAD	-1.44	-0.09	0.45	1.07
Total phenols	-1.21	-0.89	-0.55	-0.32
Lignin	-1.16	-0.93	-0.74	-0.54
Cd	0.00	1.07	0.13	-1.20
