# Supplementary material for: Effect of zinc oxide nanoparticles (nZnO) on antioxidant defense, lignin metabolism and cadmium subcellular distribution in lettuce (Lactuca sativa L) under low-dose cadmium stress (hormesis)
Source: PLoS One. 2025 Dec 4;20(12):e0337953. doi: 10.1371/journal.pone.0337953 (PMC12677453; doi:10.1371/journal.pone.0337953)
Supplement: S12 Fig — (PDF) [file pone.0337953.s012.pdf]

S12_file Fig 12

	PC1	PC2	PC3	PC4	PC5
FW	0.25	0.03	0.09	0.13	0.20
DW	0.25	0.05	0.06	0.15	0.16
GA_3	0.25	0.06	0.07	0.14	0.24
ZT	0.25	0.02	-0.03	0.18	-0.08
IAA	0.25	0.08	-0.03	0.06	-0.13
ABA	0.25	0.08	-0.01	-0.01	-0.13
MDA	-0.22	0.25	0.21	-0.02	-0.13
H_2O_2	-0.22	0.26	0.10	0.17	0.03
O_2^-	-0.21	0.29	0.14	0.24	-0.13
SOD	-0.23	0.17	0.26	-0.24	-0.32
POD	-0.23	0.22	-0.25	-0.21	0.05
CAT	0.16	0.30	0.51	-0.57	0.15
APX	0.23	0.13	0.37	0.04	0.31
PAL	0.25	0.12	-0.07	-0.00	0.02
C4H	0.22	0.28	-0.19	-0.05	-0.03
4CL	0.16	0.41	-0.26	0.03	-0.40
CAD	0.24	0.20	-0.06	-0.07	-0.26
Total phenols	0.25	0.08	-0.06	0.02	-0.22
Lignin	-0.13	0.40	-0.48	-0.20	0.54
Cd	-0.18	0.35	0.19	0.58	0.12

Principal Component Number	Eigenvalue	Eigenvalue	Percentage of Variance (%)
1	15.48	77.38	77.38
2	3.01	15.06	92.44
3	0.86	4.29	96.73
4	0.25	1.26	97.99
5	0.19	0.95	98.94
